# What Determines the Duration of War? Insights from Assessment Strategies in Animal Contests

**DOI:** 10.1371/journal.pone.0108491

**Published:** 2014-09-23

**Authors:** Mark Briffa

**Affiliations:** School of Marine Science & Engineering, Plymouth University, Plymouth, United Kingdom; University of Ottawa, Canada

## Abstract

Interstate wars and animal contests both involve disputed resources, restraint and giving up decisions. In both cases it seems illogical for the weaker side to persist in the conflict if it will eventually lose. In the case of animal contests analyses of the links between opponent power and contest duration have provided insights into what sources of information are available to fighting animals. I outline the theory of information use during animal contests and describe a statistical framework that has been used to distinguish between two strategies that individuals use to decide whether to persist or quit. I then apply this framework to the analysis of interstate wars. War duration increases with the power of winners and losers. These patterns provide no support for the idea that wars are settled on the basis of mutual assessment of capabilities but indicate that settlement is based on attrition. In contrast to most animal contests, war duration is as closely linked to the power of the winning side as to that of the losing side. Overall, this analysis highlights a number of similarities between animal contests and interstate war, indicating that both could be investigated using similar conceptual frameworks.

## Introduction

In studies of animal contest behavior, analogies with human conflict between states (wars) are frequently used. In theories about the evolution of contests, a suite of models have been based on the concept of the ‘war of attrition’ [1]–[5] where the stronger side will win through greater endurance. The war analogy is also used in empirical studies, often because a specific model is under test (e.g. [6]) or where the word has been used in a more general way [7], [8]. Here I describe how thinking about animal contests in this way has been central to the development of the field, and I outline some key similarities and differences between human warfare and animal contests. I then apply a statistical framework developed to study assessments and decision making during animal contests to data on human warfare. This will show whether war is amenable to analysis in a similar way to animal contests. Furthermore, the application of animal contest theory might provide insights into the dynamics of war.

Animal contests are direct conflicts over the ownership of limited resources. These conflicts often involve confrontations between a pair of individuals (e.g. [9], [10]) but contests can also occur between coalitions of individuals [11] or between larger groups [12]. These interactions are also described as agonistic [13] or aggressive [14] encounters or as fights [15], battles [16], [17] or wars [1]–[8]. Regardless of the terminology used, most examples are characterized by conspicuous levels of restraint. Injurious behaviors, and especially fatalities, are relatively rare and the contests are often settled using less risky activities such as signals or non-injurious trials of strength [18]. This restraint initially represented a puzzle for evolutionary biologists [19]. If natural selection produces what can be described as ‘selfish’ individuals, why should the stronger opponent in a contest show any restraint? The answer was provided by the application of a body of theory originally developed to understand the choices that humans make during conflicts of interest, namely classical game theory [20], [21]. The key insight of game theory is that the best course of action is not an absolute but is dependent on the actions of the opponent. For this reason negative frequency dependent selection, under some circumstances, can maintain a diversity of contest strategies in a population. In classical game theory, people (or organizations) are considered to be rational decision makers, who choose a course of action dependent on how their opponents are likely to behave. In evolutionary game theory natural selection, rather than individual animals, is considered to be the decision-making agent. An early evolutionary game theoretical model of contests, the Hawk-Dove game [22], [23], explains the evolution of restraint in animal contests. Here is a key parallel with interstate wars in humans. Although fatalities and material damage are clearly a feature of wars, a degree of restraint is usually present. After the war the losing polity generally continues in some form, albeit with the loss of territory, other resources or with some modified form of government imposed by the victor. As in animal contests, while there are consequences of losing, these rarely extend to the complete destruction of the losing side. Thus, regardless of whether formal peace treaties are signed, wars are often resolved by a decision on the part of the weaker side to indicate that they are willing to capitulate [24].

A related body of animal contest theory focuses on the question of how the loser should make its decision to give up. Assuming that each rival places a similar value on the disputed resource in question, contests are likely to be settled by differences in fighting ability [25], which is also referred to as Resource Holding Power [26] or Resource Holding Potential (both abbreviated to RHP). One possibility is that each opponent simply continues to fight up to a threshold which represents the maximum accrued cost it is willing or able to pay. In animal contests the costs of fighting involve time, energy and in the examples where injuries do occur, accumulated damage. Once an opponent's threshold is reached the decision to withdraw is triggered, and RHP is essentially equivalent to endurance capacity. This way of reaching a giving up decision is described by ‘War of Attrition Models’ and has been termed ‘self-assessment’ [27] as the opponent is simply using information about the level of its own threshold for giving up. Models such as the War of Attrition [2], the Asymmetric War of Attrition [5] and the Energetic War of Attrition [4] assume that fighting continues up to this threshold, without the use of information about the opponent's RHP. The Cumulative Assessment Model (CAM) [28] is based on a similar idea, but it also allows opponents to inflict direct costs on one another. Animals could certainly resolve contests in these ways, but from the perspective of the loser this is not an efficient way to make the giving up decision. It would be far better for the weaker opponent to give up before it had accrued significant costs. In order to do this it would need to assess its own RHP relative to that of the opponent. In this case each opponent would require information about itself and its rival, and hence this way of reaching a giving up decision has been termed ‘mutual-assessment’. The idea that contests are settled through mutual assessment is implicit in the Sequential Assessment Model [29], which describes opponents who sample each other's behavior in a manner analogous to statistical sampling.

There are clear parallels between these assumptions that underlie current theories about the role of information during animal contests and recent theories of interstate conflict in humans. As in the case of animal conflict, it seems logical for the weaker opponent in a war to give up as soon as it knows it is weaker [30]. Thus, similar to the idea of mutual assessment in animal contests, a key assumption of explanations for the persistence of fighting during war is that war is a process that enables each side to gather information about its relative strength. First, there is the view that warfare represents a bargaining process that allows the opponents to minimize costs by reaching a negotiated settlement as soon as possible [31], [32]. Such bargaining is assumed to occur in an iterated way where the outcomes of individual battles are coupled with subsequent rounds of negotiations, with peace offers calibrated against the outcome of the last battle. However, it has been noted that negotiations prior to the final resolution of wars are comparatively rare [33] and in some circumstances may be impeded [34], restricting the potential for the transfer of information through this route [35]. Nevertheless, battles and their outcomes may still provide a medium for information transfer between belligerent states that have divergent opinions of their own ability to win [36]. If both states believe that they are the strongest, each is unlikely to make settlement offers that are acceptable to the other. It is only when one state comes to believe that it is weaker, that a settlement offer is likely to be accepted. An alternative explanation for war duration is therefore based on the concept of attrition rather than bargaining, but the concept of attrition here has some differences to that employed in the animal contest literature. Langlois & Langlois [30] suggested that combatant states should delay negotiation for as long as possible in the hope that the opponent side will have lower capacity for persistence. In this model negotiation is absent until the end of the conflict, and the decision to give up is assumed to be driven on the one hand by the costs of remaining in the conflict and on the other by information about the costs that have been accrued by the opponent. Langlois & Langlois [30], [35] used game theoretical analyses to demonstrate that this form of attrition is the more likely to represent a stable solution compared with bargaining. They also tested for attrition-like behavior by analyzing the effect that the size of one state's military as a proportion of the total population size had on the chance of the other state's decision to give up. This measure is independent of actual military power but serves as an index of the costs that a state has accepted, potentially revealing the commitment to persistence of that state. It therefore seems logical that information about this could trigger a giving up decision in the opponent. Therefore, both types of hypothesis for war duration, bargaining and attrition, are based on the concept of information exchange between opposing sides and thus have elements in common with the models of animal contest behavior that are based on mutual assessment.

While information about commitment may be revealed by the proportionate size of the military, the role of information about military power *per se* (what each side is actually capable of) is less well understood. The distinction here is analogous to the distinction between motivation and RHP in studies of animal contests (see [37] for an example). An as yet untested hypothesis for giving-up decisions in war is that the decision to withdraw may be based on a purer form of attrition based-decision making, compared to that described in current attrition models of warfare. The weaker side's decision to give up might not be significantly influenced by any information about the opposing side. Rather it might be driven instead primarily by its own power, such that stronger losers simply last out for longer before having to give up. Below I describe an analytical framework that has been used to distinguish between mutual-assessment and this pure form of self-assessment during animal contests. In contrast with empirical studies of war duration to date, which focus on analysis of differences in motivation [30] or relative power [38], [39], this framework is based on the simultaneous analysis of winner and loser absolute power as well as the relative differences in power between opponents.

### Analysis of Resource Holding Power and contest duration: A primer

The question of how fighting animals might make the decision to withdraw is typically approached through addressing two key questions. First, what traits determine Resource Holding Power and second, how does Resource Holding Power influence the duration of contests [40]. As in the case of interstate warfare, during animal contests there are a variety of traits that might influence RHP. Larger individuals typically enjoy enhanced RHP over smaller individuals but RHP also varies with investment in weapons (e.g. the size of claws, horns, dangerous mouthparts, in both absolute terms and relative to body size) and with energy reserves (glucose, glycogen or fat reserves). In many examples of animal contest behaviour, the interaction takes place between a pair of individuals, but in some cases, such as inter-colony disputes between ants [12] or sea anemones [41], ‘multi-party’ contests [42] take place between opposing groups of individuals. In these cases the total RHP of a given side is determined by the collective RHP of the group members [16]. The total RHP advantage for the larger group may be additive or multiplicative with respect of its numerical advantage [43]. This depends on how the activities of the group are coordinated. If extra individuals are held in reserve until needed then the RHP advantage increases linearly with the disparity in numbers; if the extra individuals are all thrown into the conflict, so that each opponent individual has to fight more than one enemy, then the RHP advantage of the larger group increases as the square of the numerical advantage [43]. In interstate warfare there is a range of measures similar to those observed in animals that correlate with a state's power. An attribute commonly assumed to be indicative of power is the composite index of national capability (CINC), which is derived from a set of primary measures of power including population size, military expenditure, size of the military, energy consumption and production of iron and steel, expressed as a proportion of the total military power of all states in the world. A recent analysis [44] shows that while CINC has a strong effect on war outcomes, other factors unrelated to military power such as wealth and polity type have at best an extremely marginal influence on the outcome of war. Thus, there are a range of factors that may influence a state's ability to wage war to a greater or lesser extent. Those measures of power that demonstrably influence the chance of victory are analogous to the RHP concept used to denote fighting ability in animals.

In terms of statistical analysis, the first task then is to determine which of these measures of power actually influence the likelihood of victory. At first the question seems straightforward. We want to determine the effect of a continuous predictor on a binary (win or lose, typically coded as 0 or 1) response variable. An appropriate test would be a logistic regression analysis or generalized linear mixed model with binomial errors, if some individuals or sides appear in the data set in more than one contest. To account for non-independence of data obtained from each side in the encounter we arbitrarily designate each participant in each contest as the ‘focal’ and the other as the ‘opponent’ and calculate a composite measure that indicates the power of the focal side relative to that of the opponent. A formula for calculating relative power difference (RPD) between opposing sides is:
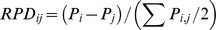
Where P  =  power of the focal individual *i* and of the opponent *j*. When *i* is stronger the value will be positive, when *j* is stronger it will be negative [40]. If the measure of power does influence the chance of winning (i.e. if it is a correlate of Resource Holding Power) there should be a significant positive effect on the chance of victory for focal the individual.

The second question is, for measures of RHP that do influence the chance of winning, do they also influence the duration of the contest? Answering this question will provide insights into whether losers make their giving up decision by comparing their own power to that of the opponent (mutual-assessment) or more directly by giving up when they reach a cost threshold that they are unwilling or unable to sustain (self-assessment). A negative correlation between duration and relative difference in RHP is expected for contests settled by mutual assessment but this could also arise spuriously [45]. If genuine mutual assessment is present we would expect to see a positive association between contest duration and the RHP of the weaker opponent coupled with a negative association with the RHP of the stronger opponent. If, on the other hand, the contest is settled by self-assessment then there should be a strongly positive association between duration and the loser's RHP and a weaker positive association with winner RHP. Taylor & Elwood [45] suggested that the best way to distinguish between assessment modes was to test for the effects of winner RHP, loser RHP and RHP difference on contest duration. An appropriate way to do this is to include all three measures as predictors of duration in a multiple regression model [45]. However, in some cases these measures of winner, loser and relative RHP might be correlated, such that problems of multicolinearity would make it impossible to analyse the data in this way. In this case, we can conduct a separate analysis for each measure, and then compare trends across the three analyses [9], [10] [40]. Interestingly, studies of animal contests show that some measures of RHP predict contest duration better than others. In the case of sea anemones for example, both body size and the size of weapons influenced the chance of winning but only weapon size influenced the duration of contests [9].

Subsequent work [46] has shown that even this method cannot fully resolve the question of mutual versus self-assessment in all cases. A positive association with loser RHP and negative association with winner RHP could still be found in the absence of mutual assessment, if the contest involves the opponents inflicting direct costs (e.g. injury or damage) on one another, so as to ‘nudge’ their opponent onwards towards their giving up threshold, as assumed in the CAM model of animal contests [28]. This is certainly a key feature of interstate warfare. Therefore, this pattern of associations cannot rule out self-assessment, if damage is a feature of the contest. On the other hand, mutual assessment can be ruled out in cases where the RHP of both opponents shows a positive association with contest duration because the diagnostic signal of mutual assessment is that duration should decline with winner RHP.

In summary there is a two-step process for analyzing animal contests that I suggest is applicable also to data on interstate warfare. The first step allows us to identify which variables might contribute to RHP and contest success. The second step allows us to rule out mutual assessment if the RHP of both winners and losers show positive associations with contest duration. This approach has been applied to examples of dyadic encounters between pairs of individuals [9], [47] but also to examples such as ants where contests occur between multiple individuals on each side [12].

Elements of the approach outlined above have already been applied to the analysis of the decision to use excess aggression during highly formalized and ritualized non-injurious inter-group contests (i.e. sport) in humans [48]. Here I apply the two-step approach to the case of the giving up decision in interstate war. I first determine which of several measures of power might influence the outcome of wars. Second I ask whether any of these measures of power, in either absolute or relative forms, influence war duration. War duration has previously been analysed from the perspective of the balance of forces, where the disparity between sides in one potential measure of power, the number of military personnel, was collapsed into a categorical variable with three levels [38]. There are no previous examples, however, where continuous measures of relative power have been analysed together with absolute measures of power, the approach that is required to test ideas about self and mutual assessment in the context of war.

## Methods

### Dataset

Data were collated from two publically available data sets maintained by the Correlates of War Project (http://www.correlatesofwar.org/). Data on war duration, winning side and losing side were obtained from the Interstate War dataset v4.0, which covers the years 1823–2003, an updated version of the data described by Ghosn et al. [49]. Data on national capabilities for the belligerents in each war were obtained from the National Material Capabilities dataset v4.0 [50]. In order to collate a data set that was amenable to the analyses described above, the following steps were performed. (1) For the interstate war dataset, data were arranged into dyads comprising the winning and losing side. Wars where a clear outcome was absent (e.g. if the war ended in stalemate or transformed into another war) were excluded from the dataset. Furthermore, to provide data comparable to analyses of animal contests, only wars that took place between a pair of individual states (rather than coalitions) were included. This yielded a dataset of N = 44 wars. (2) The duration of each war was calculated in days. (3) Five measures of power from the NMC dataset were appended to each country involved in the war. These were the primary energy consumption, *PEC*; number of military personnel, *MILPER*; total population, *TPOP*; urban population, *UPOP*; combined index of national capability, *CINC*, for the year in which they entered the war. These measures were chosen to allow a range of potential correlates of power for which relatively complete data are available. Some measures of RHP showed positive correlations, both in absolute and relative terms. For example countries with larger populations tended to have greater military expenditure. Therefore alternative approaches would have been to select one measure only for analysis or to use a multivariate approach that accounted for these correlations in a single analysis. However, to allow comparison with previous studies of both interstate warfare and animal contests, I analyse each measure of power in separate analyses. As in previous analyses of war duration [35], [51] I use power at the time of entering the war as the measure.

### Statistical methods

To determine the effect of each measure of power on the chance of victory, for each war I first assigned one side as the ‘focal’ side and the other as the ‘opponent’ side, as follows: Wars were listed by duration and the winning side was alternatively designated as focal or opponent. This ensured (a) that focal and opponent sides won an equal number of wars and (*n* = 22) and (b) that there was no difference in the mean duration of wars won by focal and opponent sides. I then calculated the relative difference between focal and opponent sides in the five potential measures of power (PEC, MILPER, TPOP, UPOP, CINC) using the formula described above. For each measure of relative power difference, I determined its effect on the likelihood of victory for the focal side using a generalized linear mixed effects model with a binomial error distribution (i.e. a mixed effects logistic regression), using Laplace parameter estimation [52], [53]. The response variable was ‘Outcome’ (1 =  victory for focal side; 0 =  victory for opponent side) and the fixed predictor variable was the particular measure of power difference under test. To account for the non-independence of data from countries that have participated in more than one war, random intercepts were included for Focal and Opponent country identity. Significance values were obtained using likelihood ratio tests to compare models that contained and excluded the fixed effects of interest. To determine the effects of winner power, loser power and relative difference in each measure of power on war duration I used general linear mixed effects models with Gaussian error distributions. Since duration data were non-normal, they were Log_10_ transformed prior to analysis. The predictors were winner power, loser power and relative difference in power. Relative difference in power was calculated as described above, except this time from the perspective of winners and losers rather than focal and opponent. As above, random intercepts were used to account for the identity of each state. An initial analysis showed that some measures of winner, loser and relative RHP were correlated. Due to these issues of multicolinearity, I therefore chose to model each measure individually, instead of including all three predictors in a single model. For significance testing of the fixed effects we used *F*-tests, calculating the degrees of freedom using the Kenward-Rogers method. All mixed effects models were calculated using the lme4 package [53] (Bates *et al*. 2013) and degrees of freedom for linear models were calculated with the pbkrtest package [54] within the R statistical computing environment, version 3.1 [55].

## Results

### Which measures of power influence the chance of victory?

**Figure 1:**
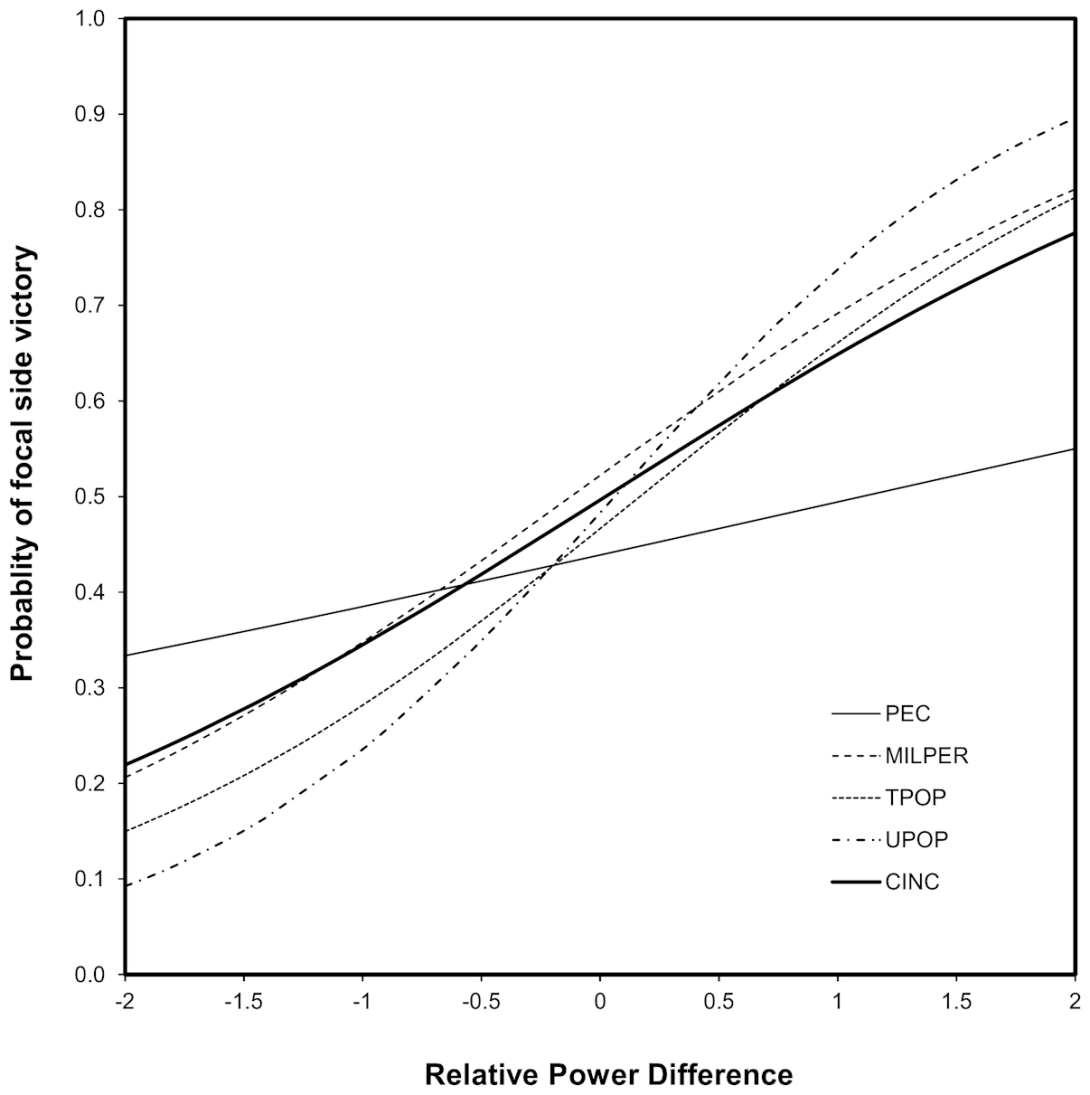
The effect of relative difference in five potential correlates of power on the chance of victory for the focal side.

The chance of the focal side winning increased with all measures of focal power relative to the opponent; CINC (χ^2^_1_ = 14.8, *P*<0.0001), size of the military (χ^2^_1_ = 10.2, *P*<0.001), primary energy consumption (χ^2^_1_ = 18.2, *P*<0.0001), total population (χ^2^_1_ = 9.3, *P*<0.005) and urban population (χ^2^_1_ = 23.1, *P*<0.0001). In the following section, the primary analysis of the effects of relative and absolute measures of power on war duration are based on the composite measure CINC. However, since all measures influenced the chance of winning (**Figure 1**) I also explore the effects of each of them on war duration.

### What determines war duration?

**Figure 2:**
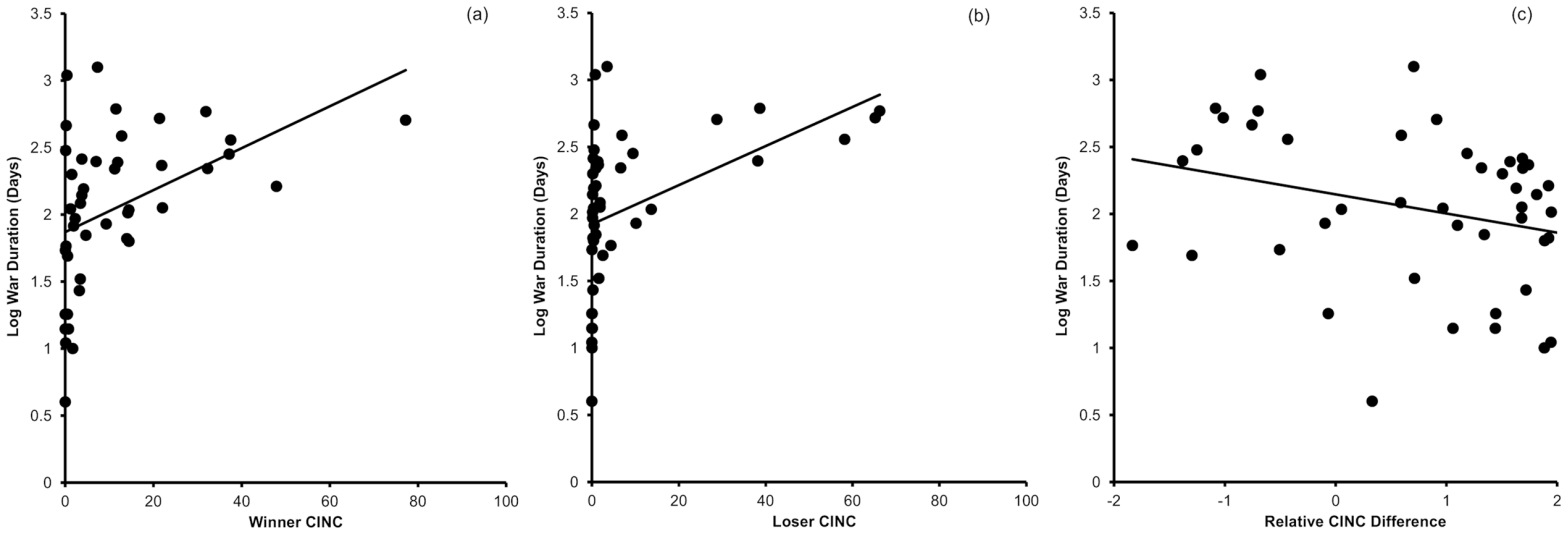
Correlations between war duration and (a) winner, (b) loser and (c) relative difference in the combined index of national capability (CINC). Regression lines fitted for illustration.

**Figure 3:**
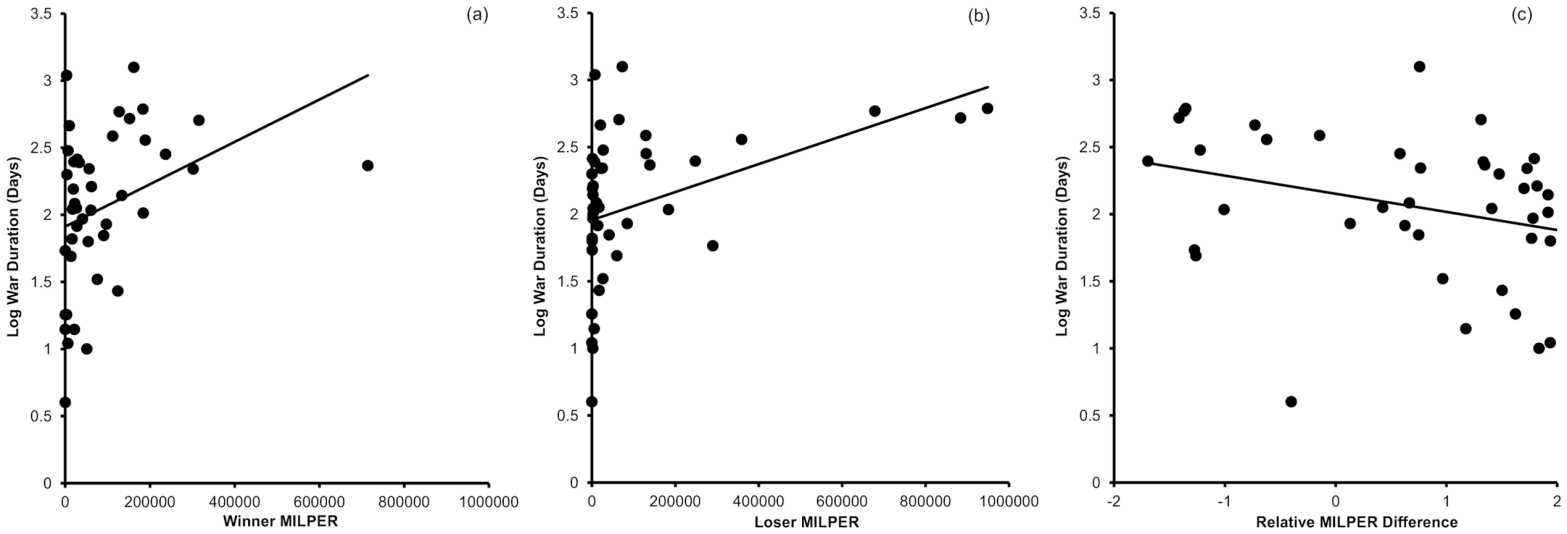
Correlations between war duration and (a) winner, (b) loser and (c) relative difference in military personnel (MILPER). Regression lines fitted for illustration.

**Figure 4:**
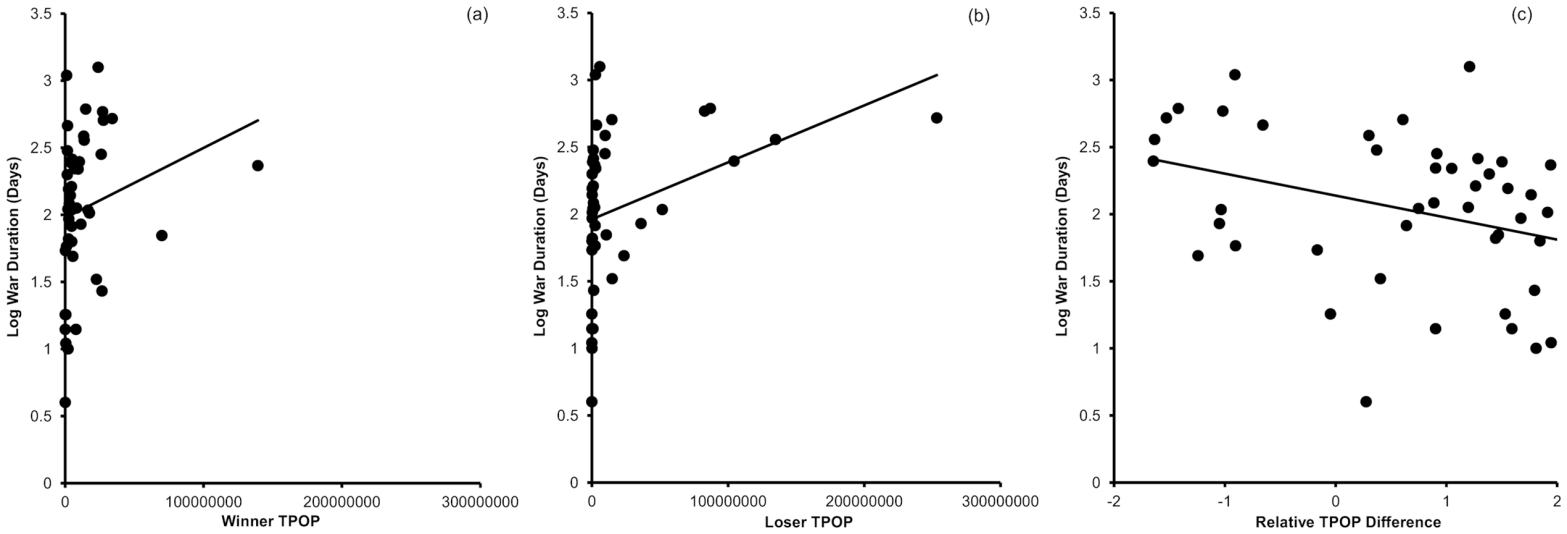
Correlations between war duration and (a) winner, (b) loser and (c) relative difference in total population size (TPOP). Regression line fitted for illustration.

War duration increased with winner CINC (*F*_1,37.5_ = 6.6, *P* = 0.015) and loser CINC (*F*_1,23.501_ = 5.9, *P* = 0.02) and there was a non-significant trend for a decrease with relative CINC (*F*_1,33.4_ = 3.2, *P* = 0.08) (**Figure 2**). War duration increased with the size of the winner's military (*F*_1,32.4_ = 9.0, *P* = 0.005) and with the size of the loser's military (*F*_1,37.4_ = 4.9, *P* = 0.03) but there was no effect of relative difference in military size (*F*_1,30.8_ = 2.1, *P* = 0.15) (**Figure 3**). War duration was not affected by the primary energy consumption of the winner (*F*_1,23.7_ = 0.05, *P* = 0.8) or the loser (*F*_1,21.5_ = 1. 3, *P* = 0.3) but duration did increase with the relative difference in energy consumption (*F*_1,24.64_ = 4.69, *P* = 0.04). Duration increased with the total population of the winning side (*F*_1,30.5_ = 7.5, *P* = 0.01) and showed a non-significant trend for an increase with the losing side's population (*F*_1,20.2_ = 3.87, *P* = 0.06) and a non-significant trend for a decrease with the relative difference in population sizes (*F*_1,32.9_ = 3.8, *P* = 0.06) (**Figure 4**). War duration was not influenced by the urban population size of winners (*F*_1,22.1_ = 1.78, *P* = 0.2) but showed a non-significant trend for increase with that of losers (*F*_1,15.89_ = 3.5, *P* = 0.08) and a non-significant trend for duration to decline with the relative difference in size between winner and loser urban populations (*F*_1,34.18_ =  3.67, *P* = 0.064).

## Discussion

A process akin to mutual assessment seems an implicit assumption of both bargaining [31], [36] and attrition [30] models currently used to explain persistence in war. An alternative hypothesis that wars could be settled primarily on the basis of self-assessment has not previously been tested. In the first set of analyses, each measure of power had a significant effect on the chance of victory. As in studies of animal contests (e.g. [10]) the strength of this effect varied between measures. Note also, that as in the case of animal contests, being the stronger opponent does not guarantee victory and in a proportion of cases the weaker individual or state might win (see [56], [57] for a discussion of why this might happen in animal contests and [58] for wars). Nevertheless on average, the stronger side is more likely to be greater in the CINC index [58] and all of the predictors in this study had a significant effect on war outcome, in the expected direction. Thus, each can be considered a valid correlate of power, analogous to the concept of RHP in animal contests. In the second set of analyses I looked at the effects of these measures of power with respect to losers, winners and the relative difference between winners and losers on war duration. Again, the strength of the results varied between analyses, dependent on the particular measure of power used. Unsurprisingly, the weakest predictor of victory, primary energy consumption was also the least informative as a predictor of contest duration. Overall, the results are similarly informative as analyses of contest duration that have been conducted using the same framework (e.g. [9], [10], [47]) and a similar pattern emerged clearly across analyses of three (CINC, size of the military and total population size) of the five correlates of power examined. First, as in a previous study [38], wars tended to be shorter when there was a large disparity in power between sides. However, as described above, this result should not be interpreted as being indicative of mutual assessment [45], a conclusion that can only be drawn in respect of the correlations between absolute measures of power and conflict duration. In-fact, the strongest driver of war duration was the power of the winner, war duration increasing with winner strength. There were also positive, although less significant, trends for duration to increase with the strength of the losing side.

Previous analyses of war have shown that the giving up decision is influenced by information about the opponent's willingness to bear costs, which is demonstrated by increasing the size of their military relative to the population as a whole [30]. This measure is independent of actual military power [30] and seems more similar to the concept of motivation in animal contests than to the concept of RHP. While information about the opponent's motivation may play a role, the current analysis provides no support for the idea that the losing side in a war reaches the decision to give up by comparing its actual fighting power (ability rather than willingness) to that of the opponent. Here we would expect at the least to see a negative association between duration and winner power with a positive association between duration and loser power, but this was not the case.

While the data are not consistent with mutual assessment they are somewhat congruent with the pattern expected for self-assessment, where we would expect war duration to increase with the power of both winners and losers. There is, however, a key difference between the prediction for self-assessment and the current results. Under self-assessment we would expect to see contest duration being primarily driven by the RHP of the loser, such that the positive association between loser RHP and duration is stronger than the positive association with the RHP of winners. Here, I found the opposite pattern with a stronger association for winners than for losers. Thus, the process differs from the pattern often reported in animal contests (e.g. [9], [47]) in that winners, rather than losers, seem more likely to make the decision to end the war. This might represent a punitive strategy whereby continued aggression discourages (or prevents) the loser from initiating future wars. Alternatively losers may be forced to settle on terms more favorable to the winner [59]. In fact punishment can also occur during conflicts of interest in animals, at least within social groups [59]–[62], and post-contest punishment may represent an understudied area of animal contest behavior. Thus, the giving up decision of states engaged in warfare clearly does not appear to be based on mutual assessment. Rather, it appears that there may be an extended form of attrition where losers use self-assessment to fight up to their maximum endurance threshold and winners may continue for longer than is strictly needed to ensure victory.

Here I have tested a fundamental assumption of models of attrition in animal contests but these models also make predictions that I have not tested here. In particular, models based on assumptions of mutual and self-assessment predict different patterns of escalation, and how these relate to the rate of cost accrual inherent in engaging in a contest [4]. When such analyses have been coupled with analysis of contest duration in animal contest studies a more complex picture often emerges. In examples including killifish [63], house crickets [10] and fallow deer [64], there appears to be a mix of assessment strategies with different modes being used at different stages of the encounter and in hermit crabs attacking and defending individuals use different sources of information about power [65]. Devising ways to quantify the rate of cost accrual and patterns of escalation and de-escalation would allow for further tests of how information is used during war. If escalation rates correlate with cost accrual rates, this would lend further support to the possibility that self-assessment is the driver of war duration.

In addition to analysis of escalation rates, there is scope to include further aspects of war that have analogues in animal contests in our analyses. These include the roles of dishonesty, third party interventions, differences in the ease of attacking and defending, and motivational differences between opponents, all of which can influence the outcome of wars [30], [66], [67] and animal contests [37], [68]–[71]. An additional area for research into wars, which has already been analyzed in animal contests, is the role of resource value. When fighting animals value a resource very highly the increase in motivation to fight might be sufficient to override differences in RHP [72]. In the case of wars over valuable territories for example, or in cases where the survival of a state is at risk (continued survival being a valuable resource), similar effects of resource value might come into play. Although some are expected to have only marginal effects on war duration [44] other factors present in wars that do not have direct analogies in contests could also be used to refine further analyses (e.g. terrain type and contiguity [30], polity type [44], [73], participation in international organizations [74], changes in leadership [75] and timing of mediation efforts [76]).

While war duration has been studied from a number of different perspectives, here I have shown how analyses developed for the study of animal contest duration and strategic decision making can be applied to duration data on war. Sun Tsu writing on the *Art of War* (ca 500 BCE) [77] stated that “*Opponents cannot exhaust you*” and furthermore that “*If you know the enemy and know yourself you need not fear the results of a hundred battles*”. Thus the potential importance of both self-assessment and mutual-assessment in the context of war have been recognized for two and a half millennia. While mutual assessment makes logical sense, in the case of animal contests recent analyses show that it may not be as pervasive as once thought [45], [46]. Similarly, mutual assessment seems very logical in the context of interstate war [30] and this assumption seems to be a common feature of previous models and analyses of power and war duration [39], [73], [78]. The current analysis, however, suggests that giving up decisions, at least in modern industrial warfare, are unlikely to be reached in this way and that some form of attrition, perhaps extended by winners, is more likely. These results thus show striking parallels between decision making during examples of contest behavior in animals and during interstate warfare. Moreover, applying the analytical framework usually used to analyze animal contests can yield new insights about interstate warfare in humans and also raises new questions about contest duration in animals, such as the possibility of extended attrition where winners might inflict additional costs on losers.
